# Exploring Medical Career Choice to Better Inform Swiss Physician Workforce Planning: Protocol for a National Cohort Study

**DOI:** 10.2196/53138

**Published:** 2024-01-17

**Authors:** Milena Abbiati, Mathieu R Nendaz, Bernard Cerutti, Monika Brodmann Mäder, Giatgen A Spinas, David Vicente Alvarez, Douglas Teodoro, Georges L Savoldelli, Nadia M Bajwa

**Affiliations:** 1 Unit of Development and Research in Medical Education Faculty of Medicine University of Geneva Geneva Switzerland; 2 Division of Internal Medicine Department of Medicine Geneva University Hospitals Geneva Switzerland; 3 Swiss Institute for Medical Education Bern Switzerland; 4 Department of Emergency Medicine University Hospital Bern Bern University Bern Switzerland; 5 Department of Radiology and Medical Informatics Faculty of Medicine University of Geneva Geneva Switzerland; 6 Division of Anesthesia Department of Anesthesiology, Clinical Pharmacology, Intensive Care and Emergency Medicine Geneva University Hospitals Geneva Switzerland; 7 Division of General Pediatrics Department of Women, Children, and Adolescents Geneva University Hospitals Geneva Switzerland

**Keywords:** career choice, medical specialty, medically underserved area, motivation, professional practice, medical students, residents, prediction model, machine learning, physician workforce

## Abstract

**Background:**

A medical student’s career choice directly influences the physician workforce shortage and the misdistribution of resources. First, individual and contextual factors related to career choice have been evaluated separately, but their interaction over time is unclear. Second, actual career choice, reasons for this choice, and the influence of national political strategies are currently unknown in Switzerland.

**Objective:**

The overall objective of this study is to better understand the process of Swiss medical students’ career choice and to predict this choice. Our specific aims will be to examine the predominately static (ie, sociodemographic and personality traits) and predominately dynamic (ie, learning context perceptions, anxiety state, motivation, and motives for career choice) variables that predict the career choice of Swiss medical school students, as well as their interaction, and to examine the evolution of Swiss medical students’ career choice and their ultimate career path, including an international comparison with French medical students.

**Methods:**

The Swiss Medical Career Choice study is a national, multi-institution, and longitudinal study in which all medical students at all medical schools in Switzerland are eligible to participate. Data will be collected over 4 years for 4 cohorts of medical students using questionnaires in years 4 and 6. We will perform a follow-up during postgraduate training year 2 for medical graduates between 2018 and 2022. We will compare the different Swiss medical schools and a French medical school (the University of Strasbourg Faculty of Medicine). We will also examine the effect of new medical master’s programs in terms of career choice and location of practice. For aim 2, in collaboration with the Swiss Institute for Medical Education, we will implement a national career choice tracking system and identify the final career choice of 2 cohorts of medical students who graduated from 4 Swiss medical schools from 2010 to 2012. We will also develop a model to predict their final career choice. Data analysis will be conducted using inferential statistics, and machine learning approaches will be used to refine the predictive model.

**Results:**

This study was funded by the Swiss National Science Foundation in January 2023. Recruitment began in May 2023. Data analysis will begin after the completion of the first cohort data collection.

**Conclusions:**

Our research will inform national stakeholders and medical schools on the prediction of students’ future career choice and on key aspects of physician workforce planning. We will identify targeted actions that may be implemented during medical school and may ultimately influence career choice and encourage the correct number of physicians in the right specialties to fulfill the needs of currently underserved regions.

**International Registered Report Identifier (IRRID):**

DERR1-10.2196/53138

## Introduction

### Background

The unbalanced distribution of medical staff is identified by the Global Health Workforce Alliance as the major public health challenge of the 21st century [1]. The equitable distribution of physicians in the appropriate specialties and in the appropriate regions challenges high-income countries that are currently dependent on foreign-trained physicians. The increasing pressure to supplement the physician workforce is because of the growing medical needs of an aging population coupled with an aging physician workforce [2,3]. A misdistribution of the physician workforce has been clearly predicted in recent statistics owing to increasing rates of part-time employment, difficulties in recruiting physicians to certain specialties, and growing gender gaps in certain specialties and places of practice [4,5].

This is particularly true in Switzerland, where life expectancy is high and 1 of 4 physicians is currently aged >60 years [2,3]. To increase the number of physicians, the federal and cantonal states have financed the creation of 4 new medical schools. However, there is little link, if any, between the physician and general population ratio and the distribution of the medical workforce; therefore, this strategy may be largely insufficient to meet the needs of the population [6,7].

Medical schools and postgraduate medical education policymaking bodies play a key role in this dynamic. They are responsible for the supply of an adequate medical workforce to meet demands for quality, quantity, and appropriate distribution of physicians among specialties and geographic areas [8-10]. The regulation of the Swiss medical postgraduate training system offers great flexibility to physician graduates regarding specialty selection, postgraduate program duration, and location. It is unique and differs from other systems in neighboring countries, where the specialty choice and training site are based on numerus clausus [2-4]. Beyond the regulation system, there is often an imbalance between students’ wishes, the need for specialists, and the positions available, leading to shortages in some specialties and high levels of competition in others [11]. Furthermore, once specialization is achieved, concentrations in certain types of practice (private outpatient rather than hospital based) and in certain geographic areas (advantaged urban rather than rural or disadvantaged urban areas) are unbalanced [12]. Consequently, there is a growing effort to better understand what factors contribute to the physician workforce shortage and misdistribution.

These factors are impacted by the societal, political, and regulatory aspects of postgraduate medical training. The Bland-Meurer theoretical framework for medical career decision-making indicates that both specialty characteristics (eg, type of practice and person oriented) and students’ career needs (eg, prestige and work-life balance) are shaped by students’ individual static (eg, demographic) and dynamic (eg, motivation) features, by life experiences, and by the values and culture of the training institution [13,14]. Studies on predominately dynamic characteristics, such as motivational factors, show that students’ specialty choice can be driven by internal motives (“intrinsic motivation”), such as personal abilities, interest in helping patients, intellectual challenge, and by external motives (“extrinsic motivation”), such as salary, status, and workload [15]. Their importance differs by specialty [16,17] and gender [18-21].

Various methods of artificial intelligence and machine learning have also been investigated to provide decision support for and predict individual career choice [22-28]. These career choice prediction and decision support studies have achieved some success at being able to predict career choice, with performance varying from 68% to 85% area under the receiver operating characteristic curve. To the best of our knowledge, only 1 study has used modern machine learning methods to predict a medical student’s career choice [29]. To date, it remains unknown how these models perform in career choice analyses in which individual and contextual data are available. Moreover, how machine learning models perform in predictive scenarios for multiple medical career choices is yet to be explored.

### Current State of Our Own Research

In our previous research, we explored the areas of intention to practice (urban and rural) of graduating students at 4 Swiss medical schools [30]. We found that 13.7% expressed an intention to practice in underserved areas (62.1% of whom intended to practice in rural areas) and 41.1% were undecided. These intentions varied from one school to another and were related to different motivational factors. Work variety and work conditions appear to be factors that might attract interested and undecided students to work in underserved areas. Among those who wished to practice in underserved areas, general practice (21.6%) was the most preferred specialty. Motivational factors influencing specialty choice were intellectual challenges, work variety, work conditions, and enthusiasm. In addition, using the same cohort at 4 Swiss medical schools, we analyzed the motivating factors that influence the choice of obstetrics and gynecology as career intentions. The results highlighted the importance of “experiential factors” and gender in this specialty choice. These findings provide useful information for targeted interventions to promote obstetrics and gynecology during undergraduate and postgraduate training by providing more hands-on experiences and improving the integration of male students and residents [31]. We also explored the degree of motivation for general practice, surgery, radiology, and psychiatry throughout the preclinical years. We specifically focused on personal and motivational individual characteristics correlated with general practice and surgical career intention [32-35].

The Swiss Medical Career Choice study is the continuation of 2 preliminary studies [12,36]. We will explore the role of students’ individual static and dynamic features as well as that of the educational organizational context and training institution culture on career choice, that is, specialty, type, and place of practice, from medical school to postgraduate training. Thus, the present proposal seeks to understand the process of Swiss medical student career choice and attempts to predict this choice. First, we will examine the static and dynamic variables that predict the career choice of Swiss medical school graduates and their interaction. Second, we will examine the evolution of Swiss medical students’ career choice and their ultimate career path.

### Study Objectives

#### Primary Objectives

The primary objectives are to determine the current career intentions of Swiss medical students (choice of specialty and type of practice) and to assess the personal and contextual factors that determine their choice.

#### Secondary Objectives

The secondary objectives are as follows:

- To assess the influence of static factors such as gender and socioeconomic status
- To assess the influence of dynamic factors such as student perceptions of medical specialties and motivation
- To examine the interactions between both types of factors
- To attempt to predict career choice based on static, dynamic, and motivational factors and to design and test data-driven methods (artificial intelligence) to predict medical students’ career choice
- To determine how career choice intentions vary during medical school within and across different medical school sites and during postgraduate training
- To determine how career choice intentions of Swiss medical school graduates have evolved over the last decade, considering the political strategies that have been put into place
- To compare how career choice intentions differ from the final choice in the Swiss nonregulated system and the French regulated system

## Methods

### Study Design

The Swiss Medical Career Choice is a 24-month longitudinal prospective national investigation implemented over 4 years (2 data collection time points) for 4 cohorts of medical students across all Swiss medical schools as well as a follow-up during postgraduate training.

For comparison purposes, this study also includes (1) a follow-up of 2 previous cohorts of medical students from Western Switzerland during postgraduate training, (2) a follow-up of the final career choice of 2 previous cohorts of medical students from 4 Swiss medical schools, and (3) a follow-up of the final career choice of 2 cohorts of medical students from Strasbourg, France.

### Participants

In Switzerland, medical school consists of a 6-year curriculum divided into 3 years of bachelor’s (basic science training) and 3 years of master’s (clinical training). For the national prospective study, eligible participants will be medical students entering year 4 (master’s 1) between 2022 and 2024 and finishing year 6 (graduates) between 2023 and 2026. The total number of eligible participants is approximately 1440 per cohort. Participants who have completed the questionnaire either in year 4 or 6 and have a response rate of >90% on the structured scale will be eligible for the follow-up study in postgraduate training year 2.

### Data Collection

Data will be collected through 4 different sources: (1) a questionnaire administered during undergraduate year 4 (master’s 1) and year 6 (master’s 2), (2) a short survey during postgraduate training year 2, (3) data extracted from the residents’ logbook of the Swiss Institute for Medical Education (SIME), and (4) exogenous contextual data extracted from the Federal Offices of Public Health and Statistics and the Swiss Medical Association (FMH).

Undergraduate participants will be invited to complete the questionnaire (approximately 30 min) during a compulsory class at the beginning of year 4 and at the end of year 6. Data will be anonymized for confidentiality reasons and for data protection issues. Participants will provide their student ID for matching purposes for the duration of the study. During the postgraduate years, participants (residents) will be invited to complete the short questionnaire (approximately 10 min) by invitation from the SIME, and relevant data will be extracted from their logbook. Data matching between the 2 data sources (undergraduate survey and data collected during residency) will be performed for each participant based on their ID number.

### Ethical Considerations

The Chair of the Cantonal Commission for Ethical Research designated this study as exempt from formal review (protocol BASEC 2020-00813) as the aim of the study aim is outside the scope of the Swiss law as defined in Article 2 of the Human Research Act (HRA).

To obtain informed consent for undergraduate data collection, eligible participants will receive an email 10 days before the survey to inform them about the research project’s main goals, the content, and the testing conditions (confidential and voluntary participation). Students who agree to participate will confirm their informed consent by marking a box on the first page of the questionnaire. Comparison cohorts of medical students consented previously to this study.

Data matching will be performed by a technical administrator who is not involved in the data analysis and interpretation. Researchers will only have access to deidentified data. Anonymous responses will be collected and stored on the secure web-based server Evasys. Survey data will be extracted from Evasys and stored in a password-protected Excel (version 21; Microsoft Corporation) file that is accessible only to the central study team. The University of Geneva standards for data handling will be followed for all data management and record keeping.

Participants will not receive compensation for completing the questionnaire.

### Measures

#### Main Outcomes

The intention of practice of the undergraduates will be assessed through a single-choice question among 6 possible options grouped into four categories: (1) hospital-based medicine (senior physician in a nonuniversity public hospital and academic and clinical career in a university hospital), (2) office-based medicine (private clinical practice in a solo practice and private clinical practice in a group practice), (3) research and teaching, and (4) undecided. The students’ specialty intentions will be gathered through a single-choice question among the 46 federal specialist titles issued by the SIME plus geriatrics, emergency medicine, and an undecided option. Specialties will be further regrouped into seven categories of intentions: (1) surgical, (2) acute care, (3) diagnostic medicine, (4) preventive medicine, (5) medical subspecialties, (6) general practice, and (7) undecided. Categories 1, 2, and 3 will be grouped together in the supracategory of “technically oriented specialties.” Categories 4, 5, and 6 will be grouped in the supracategory “person-centered specialties.” Data collected during the postgraduate training and extracted from the SIME residents’ logbook include the specialty in which the residents are currently registered, specialist title for which the resident is aiming, number of months completed in the desired specialty, and the number of months completed in different specialties. The index of change during medical school or residency will be calculated as follow: if the student does not change=0, if the student changes their intention in the same specialty category=0.5, and if the student changes for another supracategory=1. The frequency of changes will also be calculated.

#### Demographic Data

The demographic will include age, gender, nationality (Swiss, European, or other), medical school, high school diploma (scientific vs other), place of origin, marital status, mother tongue, and 2 indirect measures of students’ socioeconomic level, that is, parents’ highest educational achievement (primary, secondary, or tertiary), and parents’ profession (elementary, employee, executive, or professional) [37,38].

#### Motivational Factors

A total of 5 global motivational factors identified in the scientific literature as influencing the choice of specialty, validated by Beaulieu et al [36], will be ranked by their importance. The degree of motivation to become a surgeon as well as a general practitioner will be measured on a 6-point Likert scale: 1=very unmotivated to 6=very motivated. The global motivational factors mentioned earlier will be broken down into 12 specific motivational factors [34] influencing the choice to become a surgeon and a general practitioner (measured on a 6-point Likert scale: 1=very dissuasive to 6=very attractive). In total, 2 single-choice questions will assess (1) students’ intention to practice in medically underserved areas (yes, undecided, or no) and (2) if yes, they will be asked to specify the desired location of practice (rural, mountain, or urban areas). The desired percentage of employment will be measured as the percentage of full-time employment on a 10-point Likert scale (0=0% to 10=100%).

#### Individual Characteristics

The previous academic background will be identified through 2 items asking students to report the type of high school degree and the grade obtained. Personality traits will be measured through the NEO Five Factor Inventory, which is widely used to assess the major personality traits as described in the Big Five Model [39]. It consists of 60 items, 12 per trait, scored on 5-point Likert scales (0=strongly disagree to 4=strongly agree). Motivation will be measured through the Academic Motivation Scale, which is widely used to assess self-determinate motivation in educational settings [40]. It consists of 28 items scored on a 7-point Likert scale (1=strongly disagree to 7=strongly agree) assessing intrinsic motivation (12 items), extrinsic motivation (12 items), and amotivation (4 items). Students’ anxiety will be measured through the State-Trait Anxiety Inventory Form Y-State. Notably, we will assess state anxiety using the state anxiety subscale [41]. It consists of 20 items scored on a 4-point Likert scale (1=yes to 4=no). The total score ranges from 20 to 80. Scores are categorized into a 3-point cutoff: below 55 (average anxiety), 56 to 65 (high anxiety), and above 65 (severe anxiety). Students’ gender representation will be measured through 6 items assessing gender bias [42]. Single-item scores will be standardized into *t* scores, and a total score will be calculated.

#### Context Characteristics

Students’ perception of learning context will be measured using a brief revised version of the students’ perception of teachers in the Dundee Ready Educational Environment Measure 11-item subscale [43]. This revised version consists of 6 items scored from 0 (strongly disagree) to 4 (strongly agree) as in the original version (max score of 24). The selected questions will assess if students have identified a person they view as a role model (yes, during my medical school training; yes, but not during my medical school training; and no). If yes, 2 open-ended questions will assess (1) the function of this person and (2) in what context they met them. A total of 2 single-choice questions will assess (1) if the actual program location is the desired one and (2), if not, which location is the desired one. In total, 2 6-point Likert scale questions will assess (3) the degree of importance of the language of instruction and (4) the degree of importance of the cultural context.

Exogenous data collected from the Federal Medical and Population Statistics and the FMH include population density and medical density (of area of origin and of medical schools), number of professionals per specialty, specialist density per region, average specialist salary, and average number of working hours.

Table 1 summarizes domains, constructs, and measures by time points of data collection.

**Table 1:** Overview of domains, constructs, and measures by collection time points.

Domain and construct			Measure		Collection time point^a^				
					Master 1^b^		Master 3^c^		PGY^d^
**Main outcomes**									
	Type or category of practice	Single-choice question		✓^e^					
	Type or category of specialty	Single-choice question		✓		✓		✓	
	Rate of specialty or practice change	Homemade scale				✓		✓	
**Demographics**									
	Age, gender, and other demographics	Single-choice questions		✓		✓			
	Marital status	Single-choice question						✓	
**Motivational factors**									
	Global motives for career choice	6-point Likert scale		✓		✓		✓	
	Motivation for general practice and surgery	6-point Likert scale		✓		✓		✓	
	Motives to choose general practice or surgery	6-point Likert scale		✓		✓		✓	
	Intention to practice in underserved areas	Single-choice question		✓		✓		✓	
	Desired percentage of employment	9-point Likert scale		✓		✓		✓	
**Individual factors**									
	Previous academic background	Single-choice question		✓		✓			
	Personality	NEO-FFI^f^ [39]				✓			
	Motivation	AMS^g^ [40]		✓		✓		✓	
	Anxiety	STAI-A^h^ [41]		✓		✓		✓	
	Gender bias	Homemade scale		✓		✓		✓	
**Context**									
	Learning context	DREEM-R^i^ [43]		✓		✓		✓	
	Role modeling	Single-choice questions		✓		✓		✓	
	Master’s or training program location	Single-choice questions		✓		✓		✓	
	Exogenous data	Official records		✓		✓		✓	

^a^The questionnaires will be collected annually from 2023 to 2026 from all fourth-year and sixth-year medical students and residents of Switzerland.

^b^Master 1: fourth year of medical school.

^c^Master 3: sixth year of medical school.

^d^PGY: postgraduate year.

^e^Data collection for the associated variable at the time point.

^f^NEO-FFI: NEO Five Factor Inventory.

^g^AMS: Academic Motivation Scale.

^h^STAI-A: State-Trait Anxiety Inventory Form A.

^i^DREEM-R: Dundee Ready Educational Environment Measure.

In the postgraduate survey, the selected items of individual characteristic measures will be used, except for the State-Trait Anxiety Inventory Form A. Data collection for the Western Switzerland and French comparison cohorts is similar to that for the prospective national cohort. For the Western Switzerland cohorts, we will conduct 2 cross-sectional data collections at 2 different time points. For all comparison cohorts, we will retrieve the career choice through the residents’ logbook of the SIME and through the National Examination (Epreuves Nationales) and the National Classifying Examinations (Epreuves Classantes Nationales) official site in France.

### Data Analysis

This study adheres to the STROBE (Strengthening the Reporting of Observational Studies in Epidemiology) guidelines for cohort studies [44].

Descriptive statistics will be applied to demographics, career intention, motivational factors, and individual and contextual characteristics. A chi-square test will be used to compare intentions by medical school as well as to compare previous cohorts with the national prospective cohorts. The type I error rate will be set at .05.

To examine specific motivational factors, a principal component analysis with varimax rotation will be run to aggregate the motives out of the 12, demonstrating a frequency of >10%. Data suitability will be confirmed using the Kaiser-Meyer-Olkin index of sampling adequacy. Combined criteria (ie, scree plot, eigenvalue >1.0, and interpretability) will be used to determine the number of factors [45]. The critical value for significant factor loading will be >0.40 [46]. Each factor obtained from the principal component analysis procedure will be labeled according to its content.

Appropriate statistical tests will be applied to measure changes in motivational factors, the Academic Motivation Scale, anxiety, and contextual characteristics between different time points, such as rank-order stability (Spearman correlation *r* coefficient), the Cohen *d* effect size magnitude ratios [47], and the Reliable Change Index [48].

To examine whether the demographic, motivational factors, and individual and contextual characteristics will correlate with our main outcomes, logistic regression (odds ratio and 95% CIs) and linear regression will be used. These statistical analyses will be performed using R (version 4 or above; R Foundation for Statistical Computing) and SPSS (version 27; IBM Corp).

Following a machine learning approach, the questionnaire data and exogenous contextual data from the Federal Medical and Population Statistics and the FMH will be combined in an attempt to obtain a comprehensive view of the different medical careers within the working environment. This combined data set will then be preprocessed according to the input format of the machine learning models and will link medical students and exogenous contextual data to their career intentions. To avoid issues of data bias being injected into the algorithms, data augmentation via downsampling and oversampling strategies will be assessed to reflect equal gender distribution in the training set. For the model design and experiments, different machine learning algorithms will be investigated, in particular extreme gradient boosting [49] and CatBoost [50], which provide state-of-the-art performance for categorical data, to learn career choice patterns from the collected data set. In our experiments, the data set will be randomly divided into training (60%), development (20%), and test (20%) sets to train the model parameters and hyperparameters and to evaluate the models’ performance, respectively. Hyperparameter tuning and evaluation metrics will be computed using cross-fold validation to increase the robustness of the results. Standard classification evaluation metrics such as area under the receiver operating characteristic curve, *F*_1_-score, precision, and recall will be reported. The comparison of models’ predictive results will be measured using the McNemar statistical test. The type I error rate will be set at .05. The Shapley additive explanation method [51], a consistent, fast, and deterministic method for extracting feature contributions at the individual prediction level, will be used to identify factors impacting career choices.

### Statistical Power

Regarding the planned analyses, all the main outcomes deal with the estimation of proportions (eg, specialty choice and practice in underserved areas). Considering the smallest subgroup population of the study (2 cohorts of residents, ie, approximately n=2880) and the proportion associated with the highest variability of the estimates (*P*=.50), a sample size of 864 (ie, 2×432; refer to Table 1) would allow to estimate any proportion with a precision of +2.790% to −2.790% (95% CIs derived from the hypergeometric distribution).

## Results

The project has been peer reviewed and funded by the Swiss National Science Foundation in November 2022 with a start date of January 1, 2023, and an end date of December 31, 2026 (Multimedia Appendix 1). Data collection is currently underway, with the longitudinal cohort study having launched nationally on March 31, 2023. Because of organizational constraints, the postgraduate follow-up of historical cohorts will be launched from autumn 2023 to winter 2024. We expect to obtain preliminary results by mid-2024.

## Discussion

### Overview

This paper describes a longitudinal, prospective national investigation that will survey 4 cohorts of medical students across all Swiss medical schools as well as residents during their initial postgraduate training. The main objectives are to better understand the career choice process of Swiss medical students and to try to predict this choice.

This project will allow us to better understand the individual and contextual factors influencing Swiss medical students’ career path, from students’ intention at the end of medical school to postgraduate medical training and final specialization. This study will deepen preliminary findings on the relative influence and interaction of static and dynamic variables such as gender, work-life balance, and students’ medical specialty perceptions [12,32-36]. The fact that the study is conducted in all Swiss medical schools will also pinpoint the differences between the various Swiss medical schools and, in particular, the effects of new medical master’s programs regarding career choice and location of practice. This study will allow us to inform national stakeholders and medical schools both through prediction of students’ future choices and key aspects of physician workforce planning. Finally, by drawing comparisons with a regulated postgraduate training system such as France’s, this study will also have potential benefits at the international level.

### Strengths

To the best of our knowledge, this study is the first to investigate the personal characteristics and the evolution of students’ career choices during their medical studies and postgraduate training at a national level. Similarly, there is a lack of data regarding the follow-up from the initial intention to the definitive choice. This information is essential because better monitoring and understanding of career choice paths could help promote the management of physician resources and direct undergraduate and postgraduate interventions aimed at a better distribution of these resources [8-10].

We will provide information and prediction tools to meet demands for quality, quantity, and appropriate distribution of physicians among specialties and among geographic areas.

Using both individual and contextual data from medical students, we should be able to improve the predictive performance of the machine learning models as compared with questionnaire-only data [29]. By enabling better estimates than standard past averages, we expect that the predictive models will provide more effective support to decision makers for capacity planning. Moreover, by identifying the factors impacting career choice, decision makers will have data-driven information to support mitigation actions against specialty shortage. Finally, the predictive models may also help medical students in their career choice.

### Limitations

Difficulties in recruiting collaborators within each medical school and coordination for survey administration may hinder response rates from students and therefore impact the realization of the study’s objectives. Finally, most of the variables are self-reported and therefore subject to personal bias. However, this study will use questionnaires with established evidence to support their validity. In addition, the project has received official support from the Joint Commission of Swiss Medical Schools. This allows us to include students from all medical schools and from multiple years of study. The partnership with the Swiss postgraduate governing body, SIME, is key to enabling data collection and follow-up of subjects during the postgraduate training years. The interdisciplinary research team brings together a psychologist, a statistician, a computer engineer, and 5 medical doctors, all involved in medical education at the undergraduate and postgraduate level and holding different specialty titles.

### Conclusions

Exploring the individual and contextual factors associated with the career path of all medical students in Switzerland and the establishment of a follow-up system will provide important information for improving the quality of medical workforce planning.

We will identify targeted actions that may be implemented during medical school and may ultimately influence career choice and encourage the correct number of physicians in the right specialties to fulfill the needs of currently underserved regions. Potentially, these results could contribute to better management of the medical workforce by balancing future physician distribution and, in turn, increasing the efficiency of the health care system and meeting the needs of Swiss society.

## Appendix

Multimedia Appendix 1 Peer review report from the Swiss National Science Foundation.

## Abbreviations

FMH: Swiss Medical Association
SIME: Swiss Institute for Medical Education
STROBE: Strengthening the Reporting of Observational Studies in Epidemiology

## Data Availability

The data sets generated and/or analyzed in preparation for / prior to this study that underpin a publication are available from the corresponding author on reasonable request. The data sets generated and/or analyzed as part of this study that underpin a publication will be available in due course at the Yareta, University of Geneva data repository.
