# Supplementary material for: Exploring Medical Career Choice to Better Inform Swiss Physician Workforce Planning: Protocol for a National Cohort Study
Source: JMIR Res Protoc. 2024 Jan 17;13:e53138. doi: 10.2196/53138 (PMC10831588; doi:10.2196/53138)
Supplement: Multimedia Appendix 1 [file resprot_v13i1e53138_app1.pdf]

Review: 1

Application data

Applicant(s)

Savoldelli, Georges

Bajwa, Nadia

The Future of Swiss Physician Workforce Planning: Do Personal and Contextual Factors Predict Medical Student and Resident Career Choice?

Projects SSH

Detailed evaluation

Scientific relevance, originality and topicality

The investigators make a strong case that understanding medical student career decisions is important for medical workforce planning for Switzerland, with possible generalizability to other countries' medical education systems as well. The proposed project will advance knowledge of predictive factors and includes a reasonably broad array of potential predictors identified from prior literature reported by the authors and external investigators. The development of new methods is not as evident, as the machine learning techniques are not as clearly described. In particular, their added value beyond standard statistical methodologies is not well detailed.

As suggested above, originality is a moderate strength based on the potential for new understanding of the relative contributions of a broad range of predictors to medical student career decisions. In addition, the qualitative exploration of gendered differences in training experiences and how those differences affect career decision thought processes is appropriate and relevant as outlined in the main proposal. I would suggest focusing the proposal description on gendered differences rather than gender discrimination, however, to stay broad. Topicality of all study aims is well justified.

A very minor point is that the authors mean "predominantly" rather than "predominately" in a couple of places in the proposal.

Specific strengths

1. The topic has health care system relevance and the investigators have substantial experience in the area of study.

2. Inclusion of students from multiple schools and multiple years of matriculation enhances representativeness and generalizability.

Specific weaknesses

1. The relevance and need for the machine learning approaches is not optimally delineated.

Suitability of methods and feasibility

The main aspects of the study are not overly complex, and feasibility is good. The research plan is sensible overall although details regarding the various study cohorts and how they fit into the overall schema could be more clear. The relevance of the proposed machine learning methods could be further justified, as the plan seems amenable to standard statistical approaches for association and prediction. The qualitative exploration is well justified in the main protocol and will include a robust number of participants. Attention to participant data protections and confidentiality is good.

Specific strengths

1. Some of the data cohorts have already been initiated successfully (although this isn't entirely clear in the protocol), and this experience with metrics and survey implementation will be beneficial.

2. The application of instruments with established evidence in support of their validity is a notable strength.

3. Attention to participant data protections and confidentiality is good.

Specific weaknesses

1. It is not clear what the machine learning approach will add to more traditional statistical methods for prediction and association.

2. The integrated timeline across all aims and data sets could be more clear. The visual timeline is helpful but only reflects one part of the proposed study. For example, the retrospective student cohorts and the specific data they contain are implied to be identical to the prospective cohorts, but this not explicitly detailed.

3. As different cohorts appear to apply to different study questions, integrating knowledge across analyses will be more difficult due to inherent differences in the cohorts themselves.

4. As an observation that is not addressable, I would note that identifying predictors does not necessarily mean that modifying the predictors will change outcomes. In addition, predictors may change over time as social norms shift, etc.

5. I wondered how many Graduation Year medical students in the Swiss samples do not continue into postgraduate training in Switzerland. If this is a small proportion, the impact on the analyses should be small as well. On the other hand, if a substantial proportion of participants would be "lost to follow up" for this or other reasons, bias could result.

6. The survey instrument seems quite long. I am not sure this can be completed in 30 minutes as suggested, and even if that has been verified it is long enough that participation could be compromised. A more clear statement about investigator experiences with similar surveys and the response rates they have achieved would be helpful in this regard.

Review: 2

Application data

Applicant(s)
Savoldelli, Georges
Bajwa, Nadia

The Future of Swiss Physician Workforce Planning: Do Personal and Contextual Factors Predict Medical Student and Resident Career Choice?
Projects SSH

Detailed evaluation

Scientific relevance, originality and topicality

Le projet est pertinent et a une portée scientifique.

Il est probablement original en Suisse, pas sur le plan international où des interrogations semblables ont été explorées dans l'hémisphère Nord comme dans l' hémisphère Sud.

Specific strengths

1- La relation entre le choix de carrière des étudiants et la planification du personnel médical en Suisse

2- Le souci d'identifier des indices prédictifs dans le choix de carrière

3- L'exploration de l'évolution dans choix de carrière au cours de leur formation

4- L'hypothèse qu'une influence sur le choix de carrière aurait des retombées significatives sur les prestations de santé

Specific weaknesses

1- Le manque d'analyse globale sur les différentes causes d'une distribution défaillante des personnels de santé à travers le territoire, au delà du personnel médical, en tenant compte de l'ensemble du capital humain susceptible d'avoir une influence sur la santé.

2- Une absence de réflexion critique sur la stratégie éducationnelle de la formation médicale tout au long du cursus, tant sur le plan du contenu des études , du processus d'apprentissage et de la gouvernance académique.

3- Un questionnement sur les conditions d'exercice de la pratique médicale qui soit motivante, valorisée par le public, intégrée dans une démarche sanitaire englobant les principaux déterminants de santé dans un territoire, et donc d'une approche multidisciplinaire.

Suitability of methods and feasibility

Le projet est faisable, sans pour autant garantir qu'il réponde clairement aux attentes telles qu'exprimées dans la documentation (voir les commentaires ci-dessous).

Specific strengths

L'exploration d'une variété d'influences dans le choix de carrière , de nature personnelle, institutionnelle et environnementale.

Specific weaknesses

La méthode qui consiste à proposer un questionnaire adressé aux étudiants (self-questionnaire) sans que ceux-ci soient bien informés ou sensibilisés sur la problématique des personnels de santé, de la politique de santé et des débouchés futurs ou anticipés pour la pratique médicale, peut jeter un doute sur la pertinence de l'exploitation des réponses.

De plus, des études ont montré que les étudiants modifient leur choix de carrière en cours de formation. C'est bien démontré quand on interroge les étudiants dès leur entrée à la faculté et chaque année : ce qui peut mettre en évidence que la faculté, par son mode d' enseignement et son environnement, a une influence sur le choix, trop souvent en faveur de la médecine spécialisée . Or dans cette étude, les étudiants sont interrogés très tard (4° et 6° année), ce qui ne pourra aider significativement à identifier l'influence de la formation.

Review: 3

Application data

Applicant(s)

Savoldelli, Georges

Bajwa, Nadia

The Future of Swiss Physician Workforce Planning: Do Personal and Contextual Factors Predict Medical Student and Resident Career Choice?

Projects SSH

Detailed evaluation

Scientific relevance, originality and topicality

Most well developed nations health systems face significant workforce challenges. There are shortfalls in some worker groups and maldistributions of others. For many, the reliance on overseas trained health workers is both unsustainable and often unethical. There is a growing consensus that jurisdictions need to not only train sufficient health workers to meet their needs, now and into the future, but also need to commit to strategies to retain those workers, both within the jurisdiction, and to encourage them to work full time and at the top of their scope. Clearly, such strategies require insight into how these health worker experience their work environment and feel valued. In particular, it is important to understand what would motivate them to either leave the jurisdiction or to reduce from full-time to part time work.

It is noteworthy that the original IHI Triple Aim of care, health and cost has been extended to a quadruple aim where the fourth aim is the well-being of the workforce.

In that context, this proposed research is essential and will inform strategies to retain health workers in the workforce and to lift their well-being and productivity. In the absence of such fundamental workforce data my experience is that retention strategies are usually misdirected and unsuccessful. For example, we have found that lifestyle and autonomy are much greater influences on recent graduates' career choices than for previous generations.

Specific strengths

The study conforms to best practice in regard to surveying workforce with respect to their intentions and career decisions. The applicants have clearly taken lessons from other longitudinal studies of this sort in other countries.

Specific weaknesses

The weaknesses in the study are those of any qualitative research into individual's decision-making around careers. As far as I can see, the applicants have tried to address these as and where possible

Suitability of methods and feasibility

As cited above, the applicants have drawn on previous studies to develop a methodology, which I consider to be best practice.

Specific strengths

See above.

Specific weaknesses

Nothing significant.

Review: 4

Application data

Applicant(s)

Savoldelli, Georges

Bajwa, Nadia

The Future of Swiss Physician Workforce Planning: Do Personal and Contextual Factors Predict Medical Student and Resident Career Choice?

Projects SSH

Detailed evaluation

Scientific relevance, originality and topicality

With regards to scientific scope: the foreseen gaps in qualified medical personnel and the strategies to mitigate the effects are extremely complex issues. This proposal has been constructed with a view of collecting data from as many sources as possible so as to capture as rich and valid portrait of the dynamics affecting student career choice. This problem is not only a Swiss one but very much a global one. The promise of developing predictive models based on a longitudinal data collection strategy such as proposed here (i.e. data from now, two years ago and ten years ago) is the best that can be done given that no one knows the future. In my view, the authors propose the next best thing to the famous crystal ball of lore.

Two aspects of originality:
1) The longitudinal data collection strategy from a large sample of potential participants (N>1000). The data that will be collected will no doubt allow a greater understanding of underlying trends in career choice and professional trajectories of Swiss medical graduates that will likely go on unchanged in the future.
2) The weaving in of qualitative variables related to gender bias, work-life balance and their impact on medical career choices is an essential component in my view. Numbers don't tell the entire story. Without Work Package 3, much important and impactful insight about career choice would remain in the dark.

With regards to current relevance: the problems associated with shortages of qualified medical personnel are very acute all over the globe at the moment. There is a yet no emerging conceptualization of possible solutions to this problem in the medical education literature. Contributions stemming from the project and its predictive models will probably be trend-setting.

Specific strengths

The research team is extremely knowledgeable and accounts for much published research over the past decade. The UDREM is carving itself a well-deserved niche in medical education research.
The participation of seven 7 Swiss medical schools in the 1st Work Package is an important strength. Given the multilingual and multicultural makeup of Switzerland, the data collection strategy will be very rich and yield a nuanced portrait of career choices of all Swiss doctors over a span of 12 years.

Specific weaknesses

The added-value of machine learning is not clearly explained. Will it provide the predictive capabilities that are at the core of the proposal ? What will be the data that will be invested into building the machine learning ? To my limited knowledge, machine learning requires natural language. Where will that come from ?

Work-package 2 appears to require a little more development. As such it is unclear how the tracking system will be developed. In particular, the variable of rate of change in career choice intention is unclear, especially how it will be collected. I can understand that it will come from answers to questionnaires at different times by students, but how will these answers be generalized and used to reinforce the predictive models.

The issue of foreign trained doctors settling in Switzerland should also be accounted for. There is no mention of this when data will be collected in clinical practice settings. Are we to assume that most foreign trained doctors settle in urban university hospitals ? Are there mechanisms that assign practice settings to such doctors?

Suitability of methods and feasibility

Method selection is appropriate. Contrary to survey studies over wide populations, authors have decided to blend in qualitative data in order to explore hidden phenomena (i.e. gender discrimination, live-work balance preferences, etc.).

Given the participation of the seven medical schools and a minimum of 50% of student participants from previous studies, the feasibility of the study is assured.

Finally given the size of the work, the research team size, the four year terms seems entirely appropriate.

Specific strengths

The strategies proposed to blend the data into knowledge that will help understand the phenomena and build the predictive models.

The inter professional nature of the team, including psychologists, sociologistes, educational researchers and statisticians.

Specific weaknesses

The major risk in this project is losing too many medical school graduates who either don't respond or leave the country. Authors provide adequate mitigation strategies.

Swiss National
Science Foundation

213171, Savoldelli Georges

Projects Social Sciences and Humanities	Funded	Rejected	Total
**Proposals discussed at the meeting**	**108**	**166**	**274**
- thereof without random selection	98	156	254
- thereof in random selection group	10	10	20

Success rate[1]: 39.4%
Funding rate[2]: 35.8%

Quintile rank: 1

Your proposal falls into the highlighted group in the table.
Proposals discussed at the meeting receive a quintile rank per evaluation panel (1 indicates the top 20%).
[1] Success rate: Ratio of funded vs. total number of evaluated proposals.
[2] Funding rate: Approved amount divided by requested amount.
